# Cholesterol-Modified Amino-Pullulan Nanoparticles as a Drug Carrier: Comparative Study of Cholesterol-Modified Carboxyethyl Pullulan and Pullulan Nanoparticles

**DOI:** 10.3390/nano6090165

**Published:** 2016-09-08

**Authors:** Xiaojun Tao, Yongchao Xie, Qiufang Zhang, Ximin Qiu, Liming Yuan, Yi Wen, Min Li, Xiaoping Yang, Ting Tao, Minghui Xie, Yanwei Lv, Qinyi Wang, Xing Feng

**Affiliations:** 1 Department of Pharmacy, School of Medicine, Hunan Normal University, Changsha 410013, China; xiaojtao@126.com (X.T.); xieychao@sohu.com (Y.X.); qiuximin123@163.com (X.Q.); yuanlimingtian@126.com (L.Y.); wenyi9108@163.com (Y.W.); minli0405@126.com (M.L.); xiaoping.yang@hunnu.edu.cn (X.Y.); taotingalice@126.com (T.T.); xmh1018411891@126.com (M.X.); lvyanwei831@163.com (Y.L.); wqy9768@126.com (Q.W.); 2 Department of Pharmacology, Hubei University of Medicine, Shiyan 442000, China; zqf1112000@163.com

**Keywords:** amino pullulan, degree of substitution, surface charge, drug release, cytotoxicity

## Abstract

To search for nano-drug preparations with high efficiency in tumor treatment, we evaluated the drug-loading capacity and cell-uptake toxicity of three kinds of nanoparticles (NPs). Pullulan was grafted with ethylenediamine and hydrophobic groups to form hydrophobic cholesterol-modified amino-pullulan (CHAP) conjugates. Fourier transform infrared spectroscopy and nuclear magnetic resonance were used to identify the CHAP structure and calculate the degree of substitution of the cholesterol group. We compared three types of NPs with close cholesterol hydrophobic properties: CHAP, cholesterol-modified pullulan (CHP), and cholesterol-modified carboxylethylpullulan (CHCP), with the degree of substitution of cholesterol of 2.92%, 3.11%, and 3.46%, respectively. As compared with the two other NPs, CHAP NPs were larger, 263.9 nm, and had a positive surface charge of 7.22 mV by dynamic light-scattering measurement. CHAP NPs showed low drug-loading capacity, 12.3%, and encapsulation efficiency of 70.8%, which depended on NP hydrophobicity and was affected by surface charge. The drug release amounts of all NPs increased in the acid media, with CHAP NPs showing drug-release sensitivity with acid change. Cytotoxicity of HeLa cells was highest with mitoxantrone-loaded CHAP NPs on MTT assay. CHAP NPs may have potential as a high-efficiency drug carrier for tumor treatment.

## 1. Introduction

The low organizational specificity and severe toxicity of drugs are important factors that hinder the development of cancer treatment. Recently, a hot topic is drugs transmitted to the cancer area with high efficiency to kill cancer cells [1,2,3]. 

Nanoparticles (NPs) can load drugs to form nano-drug preparations and passively target cancer tissue because of the difference in blood-vessel characteristics in cancer tissue and rich blood flow [4,5]. Nano-drug preparations feature sustained release in the circulatory system [6,7]. Therefore, the treatment efficiency of nano-drug preparations mainly depends on cellular drug intake after they target tumor tissue. The cell uptake of the drug depends on the efficiency of NP uptake and drug loading amount. The efficiency of uptake is closely related to NP properties such as hydrophobicity, shape, size, and surface charges [8,9,10]. Positively charged NPs showed high efficiency of cell uptake [11,12]. They are largely adsorbed by the cell membrane quickly, because they have high affinity with the cell membrane whose lipid bilayer is negatively charged [13]. The NP drug-loading amount is related to the composition of the nano-material polymer [14]. Amphiphilic polymeric NPs feature a higher degree of substitution in hydrophobic groups and load more small-molecule anti-cancer drugs [15,16]. The evaluation of NP surface charge, drug-loading amount in the hydrophobic group, and cellular uptake efficiency is of great importance to screen high-efficiency nano-drug preparations. 

To design highly efficient nano-drugs, we need to understand the structure of NPs. NPs consisting of amphiphilic polymers have core-shell structures [17]. Hydrophobic groups form the hydrophobic core of the NPs as a major part of the loaded drug [18] and hydrophilic groups form the shell of NPs, improving the water-solubility of NPs loaded with a small quantity of drugs [19]. The formation of the NP spherical shape is driven by hydrophobic groups of polymers [20]. The greater the degree of substitution, the smaller the NP [21]. NP size is related to targeting and cell uptake efficiency [22,23]. The surface of polymer hydrophilic groups modifying amino derivatives or carboxylic acid derivatives, and hydrophobic groups participate in the NP self-assembly process [24,25,26]. The hydrophobic group plays a leading role in the process of self-assembly, whereas the hydrophilic amino and carboxyl groups play an important part in NP formation [16,25]. Due to the repulsive forces between NPs, amino-functionalized NPs with a positive-charge surface can prevent their aggregation [25] and increase the stability of NPs in vitro, with good targeting of tumor tissues and high cellular uptake [27,28]. Therefore, we can improve the drug curative effect by the rational design of high-drug loading with positive-charged NPs.

Pullulan has good water solubility, nontoxicity and good biocompatibility and is widely used in medicine. Because pullulan has a large amount of hydroxyl groups, it can graft small molecules such as cholesterol for hydrophobic modification and forming amphiphilic polymers [29]. As a drug carrier, cholesterol-modified pullulan (CHP) NPs can improve therapeutic efficiency because of high drug-loading and tumor-targeting [30].

The tumor-targeting function of CHP NPs results from the size, about 100 nm, and the high drug-loading amount of CHP NPs depends on the cholesterol substitution degree in polymer [31,32]. Only with an appropriate range of cholesterol substitution can polymer assemble into a core-shell structured NP [29]; within this range, the greater the substitution degree, the higher the drug-loading amount [32]. We synthesized cholesterol-modified carboxyethyl pullulan (CHCP) polymer in the early stage; under the condition that the cholesterol substitution degree is suitable for CHP polymer, carboxyethyl can affect the size and drug-loading and drug-release amount of NPs [32]. Here, we synthesized hydrophobic cholesterol-modified amino-pullulan (CHAP) polymer by new methods, identified its structure by infrared spectrum and nuclear magnetic resonance (NMR) spectroscopy, calculated the degree of substitution of the hydrophobic groups, and controlled the dosing ratio of polymer synthesis. Thus, the degree of the substitution of the hydrophobic groups was almost equal to that of the CHP and CHCP polymer synthesized in the early stage.

We prepared CHAP NPs, measured the size and electric potential by dynamic light-scattering (DLS), observed the morphology by transmission electron microscopy (TEM), measured the drug-loading amount and encapsulation efficiency, and investigated the effect of hydrophobic groups and surface charge on the formation of particle size and drug-loading amount. We compared the extracorporeal release amount and drug release amount in the acid environment of the pullulan nano-drug preparations with different surface charges and evaluated their release efficiency in extracelluar fluid of cancer cells in an acid environment after targeting cancer. We also measured the efficiency of the NP preparations in suppressing cancer cell activity and evaluated the effect of surface charge on the suppression efficiency in cancer cells.

## 2. Results and Discussion

### 2.1. CHAP Conjugate

**Figure 1:**
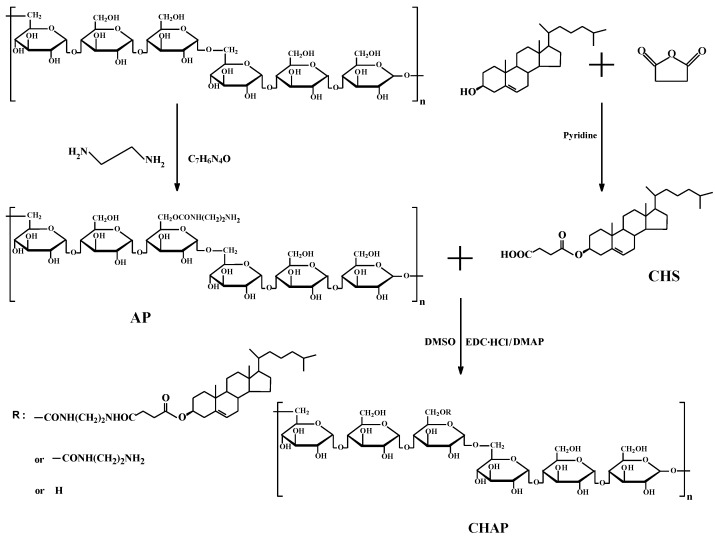
The synthesis route of the cholesterol-modified amino-pullulan (CHAP) conjugate.

Pullulan and ethylenediamine were reacted with esterification to yield amination pullulan(AP) to improve bioactivity, which was used to form an NP surface charge (Figure 1). Then cholesterol was covalently attached to AP to synthesize the CHAP conjugate with an amphiphilic property (Figure 1). 

**Figure 2:**
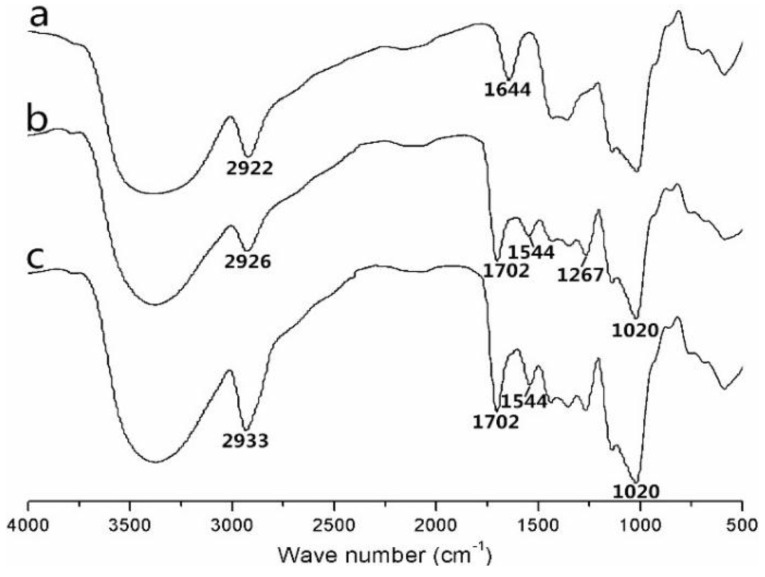
Infrared spectra of pullulan (**a**); amino-pullulan (**b**); and CHAP(**c**).

Figure 2 shows the FTIR spectra for pullulan, AP and CHAP. As compared with pullulan spectra, AP spectra was 1144 cm^−1^ (C–N stretching vibration peak), 1702 cm^−1^ (C=O stretching vibration peak, amide band I), 1544 cm^−1^ (the amide band II caused by N–H and C–N coupling), and 1267 cm^−1^ (amide band III). Therefore, the amide group was grafted to pullulan. As compared to AP spectra, CHAP spectra was 1702 cm^−1^ (C=O vibration absorption peak), and 1267 and 1020 cm^−1^ (C–O–C stretching vibration peak), so the CHAP conjugate contained an ester base. According to these characteristic peaks, CHAP was successfully synthesized by the esterification reaction.

**Figure 3:**
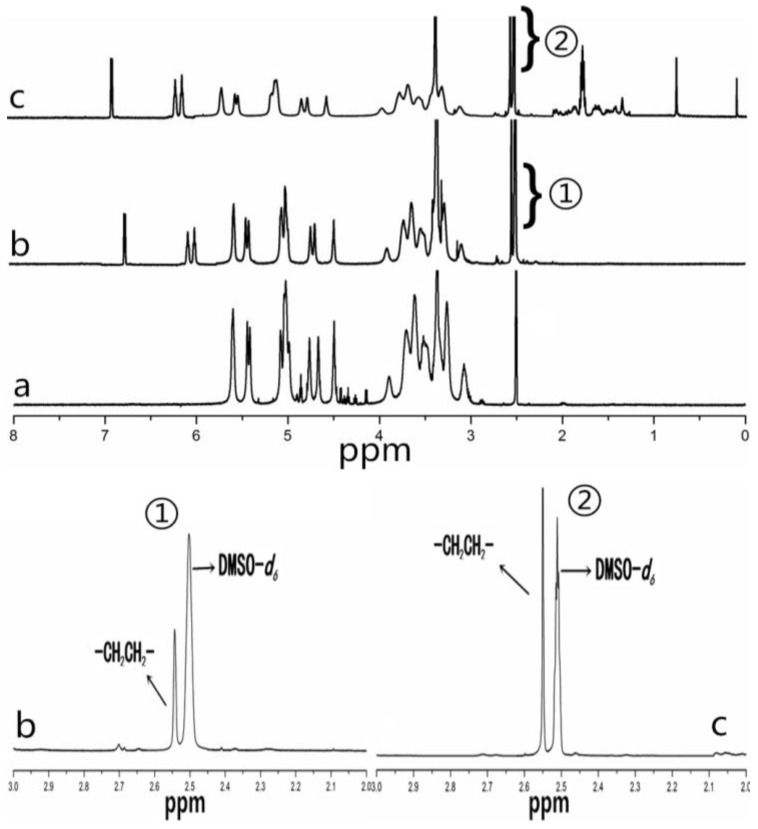
^1^H-NMR spectra for pullulan (**a**); amino-pullulan (**b**); and CHAP (**c**).

Figure 3 shows the ^1^H-NMR spectra for pullulan, AP and CHAP. As compared with pullulan, AP showed signals at 6.0 to 7.0 ppm, belonging to the amino group. The signals at 2.50 ppm were characteristic peaks for DMSO-*d_6_* and 2.55 ppm were characteristic for methylene groups (–CH_2_CH_2_–), so ethylenediamine grafted to pullulan. CHAP ^1^H-NMR spectra analysis was used to identify protons corresponding to the pullulan chain at 0 to 2.40 ppm (the H signal of cholesterol), 2.50 ppm (DMSO-*d_6_*), 2.55 ppm (methylene groups, –CH_2_CH_2_–), and 6.0–7.0 ppm (amino group). The degree of substitution of an amino group per 100 glucose units in pullulan was calculated by the ratio of methylene protons (2.55 ppm) to sugar protons (C_1_ position of α-1, 6 and α-1, 4 glycosidic bonds, 4.68 and 5.00 ppm) with the following equation [16]:

$$DS=\frac{A_{\partial 2.55}}{4(A_{\partial 4.68}+A_{\partial 5.00})}\times 100\%$$

where *A*_∂2.55_ is the spectrum area under the characteristic methylene (hydrogen) peak, and *A*_∂4.68_ and *A*_∂5.00_ are spectrum areas under characteristic proton peaks of α-1, 6 and α-1, 4 glycosidic bonds, respectively. The degree of substitution calculated from the spectra for amino groups in the AP NPs was 8.34%. The methylene groups (–CH_2_CH_2_–) of CHAP NPs included two aspects: ethylenediamine and cholesterol succinate (CHS). The degree of substitution calculated from the spectra for methylene groups (–CH_2_CH_2_–) in CHAP NPs was 11.26% and for cholesterol groups was 2.92. 

### 2.2. Property of CHAP NPs

**Figure 4:**
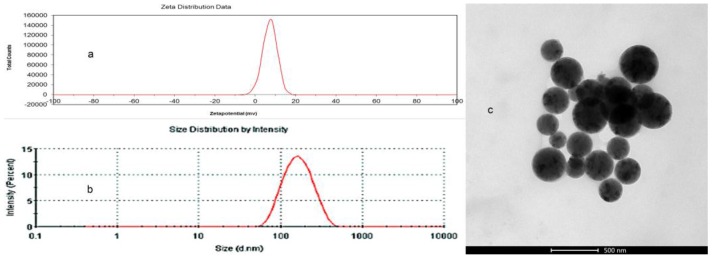
Zeta potential (**a**); size distribution (**b**); and transmission electron microscopy (**c**) of CHAP NPs.

Pullulan NPs composed of a hydrophilic pullulan shell and hydrophobic cholesterol core can load a small anticancer drug and macromolecular gene drug to form pullulan nano-drug preparations [33,34]. The preparations function in treatment, mainly in terms of NP properties such as surface charge, size, and hydrophobicity [10,11,35,36]. We previously found that CHP NPs with increasing degree of substitution with increasing cholesterol caused decreased NP size, so the formed NP size was related to the cholesterol groups [37]. In this study, we chose a degree of substitution for cholesterol in CHP, CHCP, and CHAP NPs of 3.11, 3.46 and 2.94, respectively, with close hydrophobicity. The degree of substitution of the cholesterol groups in the conjugates must occupy a suitable range for forming an NP structure. The degree of substitution for the cholesterol group in CHP NPs (2%–7%) was suitable for forming a homogeneous NP with a spherical structure, as well as CHCP and CHAP NPs, as shown by TEM in Figure 4. The figure also shows the size distribution and zeta potential of pullulan NPs with different properties. The mean size for CHAP NPs was 263.9 nm, with polydispersity index 0.138; for CHP NPs was 110.8 nm, with polydispersity index 0.267; and for CHCP NPs was 148.6 nm, with polydispersity index 0.189 [32]. The formed sizes of pullulan NPs with close hydrophobic groups differed, and carboxyl pullulan NPs and AP NPs were larger. Thus, carboxyl groups and amino groups tended to self-aggregate to form NPs, for the formation of larger-sized NPs with a loose structure in aqueous solution. CHAP NPs had zeta potential 7.22 mV as compared with −19.9 mV for CHCP NPs and −1.21 mV for CHP NPs [32]. Thus, positive-charged amino groups and negative-charged carboxyethyl groups contributed to particle surface charges. 

### 2.3. Mitoxantrone (MTO) Loading of Pullulan NPs with Different Surface Charge 

Pullulan polymer NPs contain a hydrophilic sugar chain as an external shell and hydrophobic cholesterol groups as an interior core, which can load small molecular drugs to form nano-drug preparations [38]. The treatment efficiency of nano-drug preparations depends on the drug loading amount, which is closely related to the degree of substitution of hydrophobic groups in the polymer [32]. The CHP NPs with a higher degree of substitution of cholesterol groups can load more MTO [32]. We chose pullulan NPs with different surface charge and a close degree of substitution of cholesterol groups to evaluate the drug-loading amount. MTO was loaded into pullulan NPs at a ratio of 4 mg MTO to 20 mg pullulan NPs by the dialysis method. Table 1 shows encapsulation efficiency for CHP, CHCP, and CHAP NPs of 75.2%, 72.4%, and 70.8%, and loading capacity of 13.3%, 12.7%, and 12.3%, respectively. CHCP NPs with cholesterol substitution 3.46 could form negative-charge NPs with drug-loading capacity of 12.7% as compared with 13.3% for CHP NPs. CHAP NPs with cholesterol substitution 2.92 could form positively charged NPs with drug-loading capacity of 12.3% as compared with 13.3% and 12.7% for CHP NPs and CHCP NPs. Thus, besides the hydrophobic cholesterol groups in the NPs deciding the drug-loading behavior in the formed NPs, the carboxyethyl group or amino groups also affected the drug-loading amount. Pullulan NPs showed little change of surface charge after drugs were loaded, shown in Table 1 as zeta potential −1.21 ± 0.12, –19.9 ± 0.23, and 7.22 ± 0.18 mV for CHP, CHCP and CHAP NPs, respectively.

### 2.4. Effect of Media pH Value on Drug Release of Pullulan NPs with Different Surface Charge

**Figure 5:**
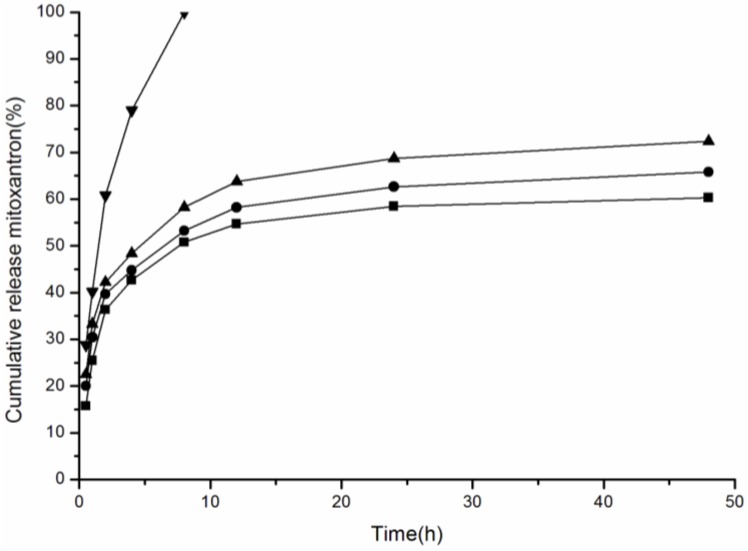
The mitoxantrone (MTO) release of nanoparticles in phosphate buffered saline (PBS) at 37 °C in vitro (▼ free mitoxantrone, ■ CHP, ● CHCP, ▲ CHAP).

Drug release of NPs is related to NP hydrophobicity, surface charge, and release media pH [32]. In this study, we measured the drug release of CHAP NPs and studied the drug release of pullulan NPs with close hydrophobicity and different surface charge. In Figure 5, free MTO has a fast release, with 99.94% of the total drug released after 8 h. Drug release of pullulan NPs with different surface charge all showed sustained release. The drug release in 48 h included two steps: fast release within 8 h and slow release after 8 h, attributed to the drug-loading pattern in pullulan NPs. The total drug release amount was 60.28%, 65.78%, and 72.36% for CHP, CHCP and CHAP NPs, respectively. With close hydrophobicity, pullulan NPs with positive surface charge showed greater drug release. 

**Figure 6:**
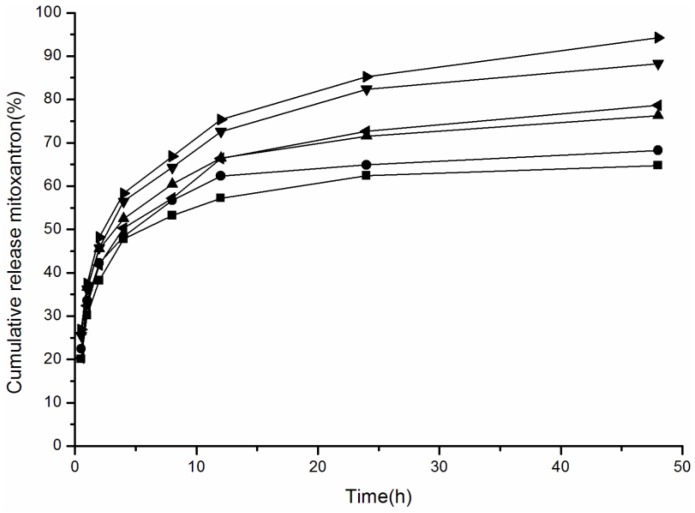
MTO release from NPs in phosphate buffered saline buffer (pH 6.8) at 37 °C in vitro and acetate buffer (pH 4.0) (pH 6.8: ■ CHP, ● CHCP, ▲ CHAP; pH 4.0: ◄ CHCP, ▼ CHP, ► CHAP).

When nano-drug preparations are targeted to the cancer tissue, the drug release amount increases substantially under the acidic environment of the cancer extracellular fluid, which causes the release at the cancer location to kill cancer cells with high efficiency [39,40,41]. Therefore, for cancer treatment, we must study the drug release of NPs with acid pH. We measured the drug release of CHAP NPs with weak and strong acid pH and evaluated the media pH effect on drug release of pullulan NPs with different surface charge. Figure 6 shows the drug release of CHAP, CHCP and CHP NPs in release media at pH 6.8 and 4.0. In release media at pH 6.8, the drug release amount was 64.78%, 68.25% and 76.27% for CHP, CHCP and CHAP NPs, respectively, at 48 h. The drug release was fast and substantially increased with release media at pH 4.0: 88.24%, 78.67%, and 94.26% for CHP, CHCP and CHAP NPs, respectively, at 48 h. Therefore, drug release in the pullulan NPs favored an acidic environment. The drug release amount was enhanced with increasing acid intensity. Although the three kinds of NPs all showed greater drug release with increasing acid intensity, the drug release sensitivity differed by acidity. Compared to CHP NPs with drug release from 64.78% to 88.24%, CHCP and CHAP NPs showed drug release from 68.25% to 78.67% and 76.27% to 94.26%, respectively. CHAP NPs showed more drug release than other two NP types, and the sensitivity was stronger than with negative-charged CHCP NPs. Self-aggregated NPs are degraded under strong acid release media (pH 4.0) [41,42,43]. Positive-charged CHAP NPs may be degraded more easily than negative-charged CHCP NPs, for a greater drug release amount with a strong acid environment. Comparative analysis of drug release of NPs with different surface charge is needed for designing nano-drug preparations with highly efficient tumor treatment. 

### 2.5. In Vitro Cytotoxicity 

**Figure 7:**
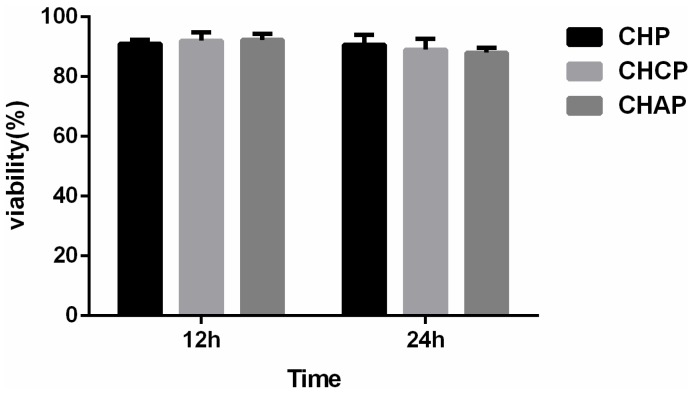
In vitro viability of HeLa cells with NPs at 12 and 24 h. Data are mean ± SD (*n* = 3).

**Figure 8:**
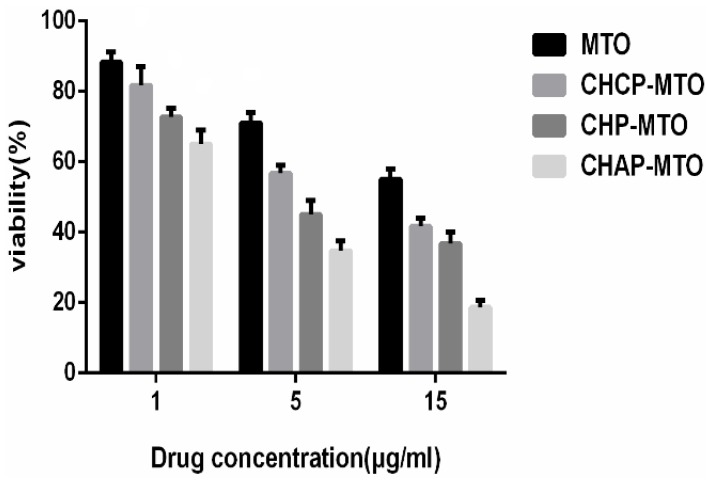
In vitro viability of HeLa cells with MTO alone and MTO-loaded NPs at 24 h. Data are mean ± SD (*n* = 3).

After NPs target tumor tissue, the drug is partly released in the extracellular fluid and the treatment function is displayed by cell uptake with free drug. However, tumor treatment mainly depends on cell endocytosis of nano-drug preparations [44]. Cell endocytosis efficiency of nano-drug preparations was evaluated for the cell proliferation, which can be measured by MTT assay. Cytotoxicity was measured by MTT cell viability assay in HeLa cells. HeLa cells were incubated with MTO-loaded NPs at a dose equivalent to that of free MTO. Three kinds of blank NPs showed no significant cytotoxicity at the highest concentration (2 mg/mL) in cells after 12- and 24-h incubation (Figure 7). With the same dose treatment, cell viability decreased with increasing concentration of MTO-loaded NPs from 1 to 15 mg/mL as well as MTO alone (Figure 8). The cytotoxicity of MTO-loaded NPs and free MTO increased with increasing MTO concentration. Growth of HeLa cells was inhibited more with MTO-loaded NPs than free MTO. MTO-loaded CHAP NPs showed stronger cytotoxicity than other two drug-loaded NPs, and cell inhibition rates were 35%, 65.4%, and 81.4% with 1, 5, and 15 mg/mL treatment, respectively. Cell inhibition testing suggested that CHAP NPs as a drug carrier may have high tumor treatment efficiency with improved drug loading. 

## 3. Discussion

Nano-drug formulations have aroused great interest because they can significantly improve the efficacy of anti-cancer drugs by targeting cancer tissue and reducing toxicity [45,46]. Research into nano-drug preparations has made great progress, but only a few reports describe the implementation process and the factors that affect efficiency in nano-drug treatment. The therapeutic efficacy of nano-drug preparations includes two steps: the targeted tissue and penetrating the cell membrane. 

The NP target site is closely related to the size of particles and protein coating [22,47]. For example, the nano-level particle size in the 100- to 200-nm NP size can passively target cancer tissue. The function of nano-drug preparations and the effects of proteins are inseparable. For example, after using polysorbate to modify the surface of NPs, nano-drug preparations can deliver drugs to brain tissue through the blood–brain barrier, for brain targeting, when mediated by apolipoproteins [48]. NPs can be swallowed by the reticulo-endothelial system of liver and passively target liver tissue mainly because they can absorb tropin and integrin in the blood circulation and lead to accumulated Kupffer cells in liver [49].

After the NP surface is modified by PEG, because of the steric hindrance of the hydrophilic-chain PEG, the absorption of opsonins and integrins is blocked, thereby weakening the phagocytosis of the liver reticuloendothelium system [50]. Because normal blood vessels have a barrier function under non-pathological circumstances, particles of nano-size cannot penetrate the blood capillaries, so PEG-modified NPs can circulate in vivo for a long time, for a long-lasting release function. Thus, protein-absorbed NPs have great importance in the targeting function of NPs; however, the targeting character of NPs ultimately depends on NP characteristics, such as material ingredients and surface quality, which decide the kind and amount of the protein absorbed [51,52]. The aggregation and nonspecific adsorption of cationic nanocarriers with serum albumin in the bloodstream stimulate clearance by phagocytic cells and the reticuloendothelial system. 

**Figure 9:**
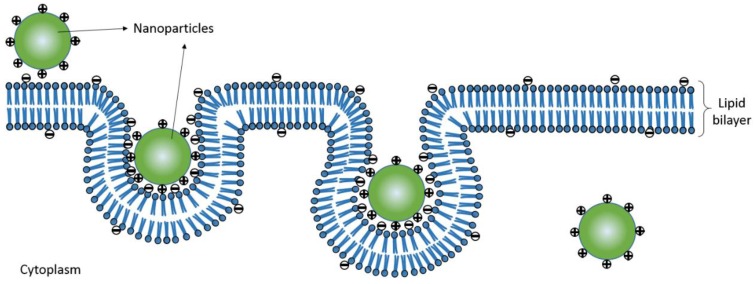
The cell uptake illustration of nanoparticles with surface positive charge.

**Figure 10:**
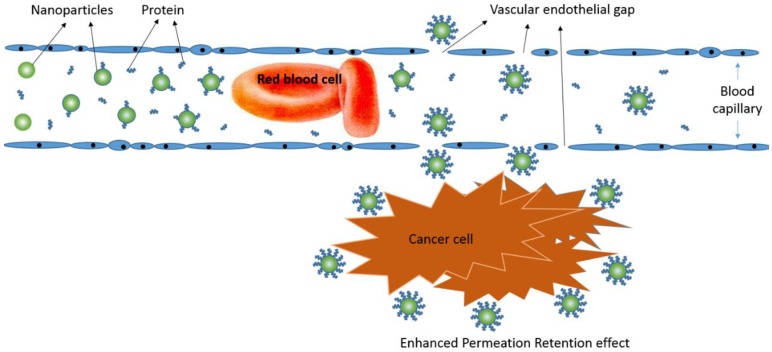
Illustration of movement of NPs from blood to targeted tissue.

The cellular uptake efficiency of positive-charged NPs is higher than neutral NPs and negative-charged NPs because the cell membrane phospholipid bilayer is negatively charged, which helps with positive-charged NP adsorption, then endocytosis occurs under cell membrane protein mediation [53] (Figure 9). However, before the positive-charged NPs enter the tumor cell, they can be adsorbed by plasma proteins and the electric charges are cleared by shielding the adsorption of negative-charged protein, finally causing lower cellular uptake efficiency [54]. When NPs target the tumor tissue and reach the cell, the cells meet NPs absorbed with protein [55]. Nano-protein coronas touch the cell membrane directly, so the charge that NPs take will not affect cell uptake directly (Figure 10). 

Therefore, the therapeutic efficiency of positive-charged NPs can avoid the presence of drugs in circulation, for highly efficient cell uptake. The therapeutic effects can be enhanced by providing drugs locally as in intravesical infusion in superficial bladder cancer. CHAP is a kind of positive-charged NP with a polysaccharide cloak. It can be delivered to tumor locations by irrigating the bladder. Polysaccharide can enhance the tissue adhesion of NPs and the fixation of tumor location. The positive charge can enhance cell adhesion and the efficient uptake of drugs.

When in the acidic environment around the tumor, the amount of drug released by CHAP NPs is greater than that released by negative-charged and neutral NPs. The release of free drug and the cell uptake efficiency all indicate that CHAP NPs may have potential as an excellent carrier of intravesical therapy in cancer. 

## 4. Materials and Methods

### 4.1. Materials

CHP and CHCP NPs were synthesized as described in [16,37]. 3.11 cholesterol groups were grafted to glucose moieties of pullulan per 100 units in CHP NPs. The degree of substitution for carboxyethyl and cholesterol groups per 100 glucose units in CHCP NPs was 10.13% and 3.14%. *N*,*N*-carbonyldiimidazole was from Shanghai YuanYe Biological Technology (Shanghai, China). Ethylenediamine was from Tianjin HengXing Chemical Reagent (Tianjin, China). Mitoxantrone (MTO) was from Beijing Huafeng United Technology (Beijing, China). Diphenyltetrazolium bromide (MTT) was from Sigma Co. (St. Louis, MO, USA). All other chemical reagents were of analytical grade and obtained from Changsha Huicheng Commerce (Changsha, China).

### 4.2. Synthesis of CHAP NPs

Synthesis of amination pullulan (AP): 1.80 g pulullan and 1.00 g *N*,*N*-carbonyldiimidazole, were dissolved in 100 mL dewatered dimethyl sulfoxide (DMSO); after heating and stirring in a 50 °C oil bath for 4 h, 3.60 g ethylenediamine was added for heating and stirring for 24 h, then the reaction was stopped. When the reaction liquid cooled to room temperature, it was placed in a 4000 interception dialysis bag, with double-distilled water for dialysis for 1 day, then lyophilization. A light yellow solid was the product of amino pullulan.

Synthesis of CHAP: Cholesterol succinate (CHS) was synthesized as described in [26]. An amount of 2.00 g AP was dissolved in an appropriate amount of DMSO. Then 1.06 g CHS, 0.505 g EDC•HCl and 0.268 g DMAP was dissolved in an appropriate amount of DMSO. The above two groups of reagents were mixed and activated for 1 h at room temperature and reacted at 50 °C in a heated oil bath pot for 48 h; the reaction was stopped and cooled to room temperature; then dropped into the right amount of anhydrous ethanol by stirring constantly to precipitate a white solid and obtained by suction filtration several times with a moderate amount of anhydrous ethanol, ethyl ether, and tetrahydrofuran wash products; the products were placed in a blast dryer at 50 °C. The end product was a light yellow solid.

### 4.3. Fourier Transform Infrared (FTIR) Spectroscopy and NMR Spectroscopy

The FTIR spectra for pullulan, AP and CHAP were obtained as KBr pellets for FTIR spectroscopy (Nicolet NEXUS 470-ESP, Thermo Fisher Scientific Inc., Waltham, MA, USA). The chemical structure of pullulan, AP and CHAP was confirmed by 500 MHz ^1^H-NMR, using DMSO-*d6* as the solvents. The degree of substitution, defined as number of cholesterol and amino residues per 100 glucose units of CHAP, was determined by ^1^H-NMR.

### 4.4. Preparation and Characterization of Pullulan NPs

CHAP NPs were prepared by the dialysis method [37]. Briefly, CHAP was dissolved in 1 mL dimethyl sulfoxide. To form NPs, the solution was injected in a dialysis bag for dialysis for 24 h to get rid of dimethyl sulfoxide. The solution of CHAP NPs was screened with a membrane filter (pore size: 0.45 µm, Millipore, Boston, MA, USA) to remove the larger sizes of the aggregated CHAP NPs .The size distribution and zeta potential of obtained particles were determined by DLS (Zetasizer 3000 HS, Malvern Instruments, Malvern, UK) at 11.4 V/cm, 13.0 mA. The solution of CHAP NPs (1.0 mg/mL) was dropped onto a copper grid, followed by the application of phosphotungstic acid (2%) negative stain, and morphology was observed by TEM (Tecnai G^2^ 20 S-Twin, FEI Hong Kong Inc., Hong Kong, China) at accelerating voltage 80 kV. CHP and CHCP NPs were prepared and characterized by the same method. 

### 4.5. Preparation and Characterization of MTO-Loaded NPs

Drug-loaded pullulan NPs were prepared as described [32]. Briefly, 4 mg MTO and 20 mg NPs were dissolved in 20 mL DMSO, and an amount of trethylamine was used as the propellant to help CHAP NPs with higher drug loading. MTO-loaded NPs were obtained by dialysis (weight cutoff, 12–14 kDa, Millipore) for 9 h to remove DMSO and superfluous drug. The zeta potential of the obtained drug-loaded CHP, CHCP and CHAP NPs were measured by DLS. Drug loading content (%) and efficiency (%) of CHAP NPs were calculated with following formula [37]:

Drug loading efficiency (%) = NP drug-loading amount/initially added drug amount × 100%
Loading content (%) = NP drug-loading amount/NP drug carrier amount × 100%

### 4.6. In Vitro Drug Release of Pullulan NPs with Different Properties

The solution of MTO-loaded CHAP NPs was put in Visking dialysis bag and dialyzed in the release media of phosphate buffered saline at 37 °C. Then, 2 mL of the solution of release media was collected and substituted with an equal volume of the fresh solution at pre-defined time intervals (Ti). The drug release amount was measured by UV spectrometer (UV-384 plus, Molecular Devices, Thermo Fisher Scientific Inc., Waltham, MA, USA), and The percentage rate of drug release (*Q*%) was measured as follows [30].

$$Q\%=(C_{n}\times V+V_{n}\sum_{i=0}^{i=n}C_{i})/W_{drug-loading}$$

where *C*_n_ is the sample concentration at *T*_n_, *V* is the total volume of release medium, *V*_i_ is the sample volume at *T*_i_, *C*_i_ is the sample concentration at *T*_i_ (both *V*_0_ and *C*_0_ were equal to zero), and *T*_n_ is the sampling at the *N*_th_ time. The drug release features of CHP and CHCP NPs were also collected to compare with the drug release of CHAP NPs.

### 4.7. In Vitro Drug Release of Pullulan NPs in Release Media with Different pH

In phosphate buffered saline (pH 6.8) and acetate buffer (pH 4.0), we measured MTO release amount in vitro by a dialysis method. Briefly, MTO-loaded CHAP NPs was placed into a dialysis tube (weight cutoff, 12–14 kDa, Millipore) and dialyzed against the release media at 37± 0.2 °C in an air-bath shaker at 100 rpm. Then, the release percentage was calculated with collection of drug release amount as described previously. The drug release amount from CHP NPs was collected in media at different pH to compare with that from CHCP NPs.

### 4.8. In Vitro Cytotoxicity

The cytotoxicity of blank NPs, MTO-loaded NPs and free MTO was determined by measuring the inhibition of cell growth by tetrazolium dye (MTT) assay as described [56]. Briefly, HeLa cells were seeded at 5 × 10^3^ cells/well in 96-well plates. After 12 and 24 h incubation, cells were treated with a concentration of blank NPs with different surface charge (2 mg/mL), MTO-loaded NPs and free MTO in serum-free medium for 24 h, then with MTT solution (20 μL, 5 mg/mL in PBS) for 4 h at 37 °C. The resulting formazan was dissolved in DMSO (150 μL) and measured at 490 nm by using a microplate reader. The cellular growth inhibition was calculated as follows:

$$\mathrm{inhibition}\text{\%}=(1-\frac{Test-Blank}{Control-Blank})\times 100\%$$

We used untreated cells as the control (100% survival) and non-cell wells as the blank to substrate solvent absorbance. 

## 5. Conclusions

Pullulan was grafted with amino-ethyl groups and cholesterol groups to form CHAP NPs with positive surface charge, which was compared to two other types of NPs with close cholesterol hydrophobic properties: CHP and CHCP. The amino-ethyl groups affected the NP size formed and directly affected the formation of the positive surface charge. The drug-loading amount depended on NP hydrophobic groups and was affected by NP surface charge. NPs with positive or negative surface charge showed more drug release than NPs with neutral surface charge. The drug release amounts of NPs were all increased in the acid media and increased with increasing acidity. However, the drug release of CHAP NPs was sensitive to acid change, and the drug release amount in strong acid media was higher with CHAP NPs than the two other NPs. Drug-loaded CHAP NPs showed high cytotoxicity, so they may have potential as a drug carrier for tumor treatment.

## Figures and Tables

**Table 1:** Characteristics of mitoxantrone-loaded pullulan NPs with different surface charge.

Sample	D/C (*w*/*w*) ^a^	EE (%) ^b^	LC (%) ^c^	ζ (mV) ^d^
CHP	1/5	75.2 ± 1.94	13.3 ± 0.22	−1.21 ± 0.12
CHCP	1/5	72.4 ± 1.72	12.7 ± 0.20	−19.9 ± 0.23
CHNP	1/5	70.8 ± 1.68	12.3 ± 0.20	7.22 ± 0.18

^a^ Weight ratio of drug and carrier (mg/mg); ^b^ encapsulation efficiency determined by ultraviolet spectrophotometry at 608 nm; ^c^ loading capacity determined by ultraviolet spectrophotometry at 608 nm; ^d^ zeta potential of drug-loaded pullulan NPs measured by dynamic laser light-scattering.
